# Modified Two-Surgeon Technique for Laparoscopic Liver Resection

**DOI:** 10.7759/cureus.23528

**Published:** 2022-03-27

**Authors:** Takahisa Fujikawa, Masatoshi Kajiwara

**Affiliations:** 1 Surgery, Kokura Memorial Hospital, Kitakyushu, JPN; 2 Gastroenterological Surgery, Faculty of Medicine, Fukuoka University, Fukuoka, JPN

**Keywords:** role sharing, saline-linked electrocautery, two-surgeon technique, liver parenchymal transection, laparoscopic liver resection

## Abstract

While minimizing intraoperative blood loss during liver resection is one of the most important tasks, it is more difficult to control the refractory bleeding during laparoscopic liver resection than with an open approach. We herein provide a modification of the two-surgeon technique that enables laparoscopic liver parenchymal transection to be performed as quickly and securely as open liver resection. To achieve proper "role sharing," the "transection mode" and the "hemostatic mode" are independent sets in place in this procedure, and these modes are switched rigidly according to the surgical field condition. By thoroughly sharing the roles, rapid laparoscopic liver parenchymal transection comparable to open liver resection can be accomplished. The present modified approach achieves satisfactory transection and hemostasis of the liver parenchyma and is also advantageous for teaching young surgeons and the entire surgical team.

## Introduction

The safety of liver resection has improved substantially in recent decades [1,2], and thanks to the introduction of numerous energy devices and procedures, laparoscopic liver resection (LLR) is now one of the favored options [3-5]. One of the most important tasks during liver resection is minimizing intraoperative surgical blood loss, and improvements in several technical aspects, such as the liver hanging maneuver, the Pringle maneuver (intermittent hepatic vascular inflow occlusion), or the "two-surgeon technique," have been reported [6-10].

The two-surgeon technique was first utilized for open liver resections, and it was found to reduce operative time, surgical blood loss, and postoperative bile leakage [6-8]. The need for "role sharing" between the primary and secondary surgeons is critical in this procedure. The primary surgeon dissects the hepatic parenchyma, while the secondary surgeon employs a saline-linked cautery to focus on hemostasis (SLiC). However, performing these procedures at the same time during LLR is difficult, and in the event of bleeding from the deep parenchymal fissure or a major hepatic vein, the secondary surgeon must collaborate with the primary surgeon.

We herein provide a modified two-surgeon technique for laparoscopic liver parenchymal transection that can be performed as quickly and safely as open-fashioned hepatectomy.

## Technical report

Role sharing in the two-surgeon technique

The "transection mode" and the "hemostatic mode" are independent sets in place in our modified procedure to ensure proper "role sharing," and these modes are switched strictly according to the surgical field situation.

**Figure 1:**
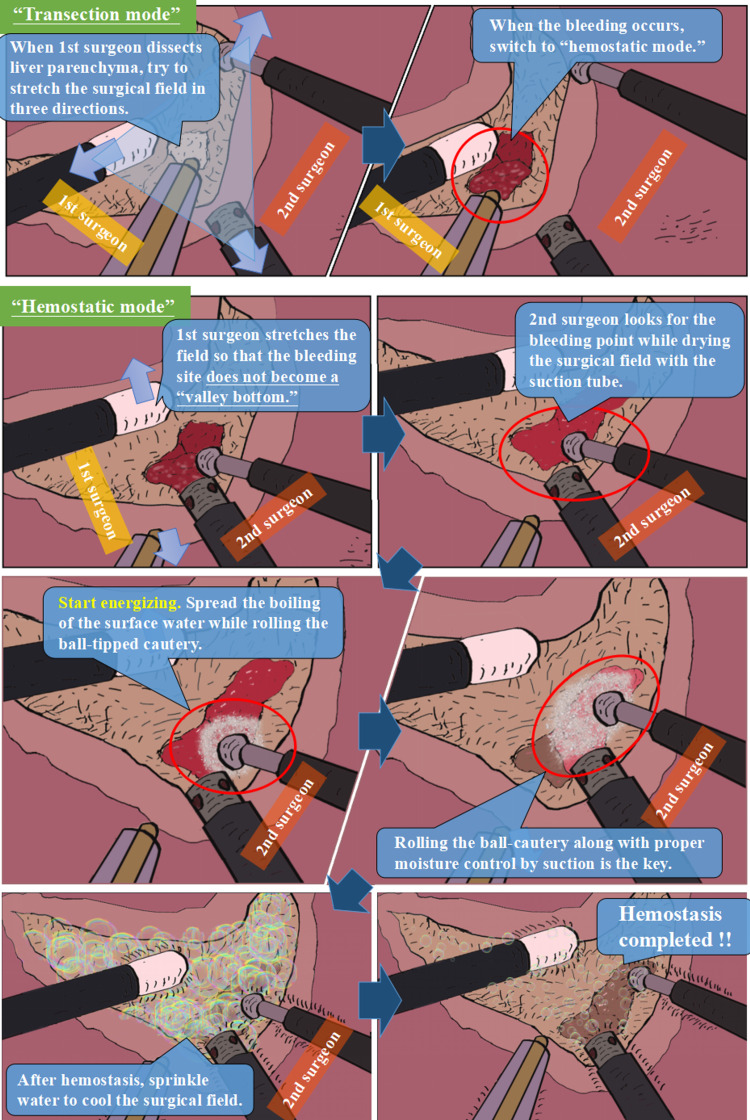
The outline of the modified “two-surgeon technique” in laparoscopic liver resection. In the "transection mode," the primary surgeon dissects the parenchyma using the Cavitron ultrasonic surgical aspirator while the secondary surgeon stretches the surgical field using ball-tipped electrocautery and suction tube. The mode is quickly shifted to "hemostatic mode" if bleeding from the deep parenchymal fissure begins. During the "hemostatic mode," the primary surgeon stretches the surgical field to help the secondary surgeon achieve hemostasis. The surgical area is stretched sufficiently to prevent the bleeding location from becoming a "valley bottom," and the secondary surgeon uses a hemostatic approach appropriate for the bleeding level.

In "transection mode," the primary surgeon dissects the parenchyma with a Cavitron ultrasonic surgical aspirator (CUSA) or laparoscopic coagulating shears, while the secondary surgeon stretches the surgical field well using a ball-tipped SLiC and a suction tube. When the primary surgeon dissects the liver parenchyma, the two surgeons collaborate to stretch the surgical field in three directions ("tissue triangulation"). The secondary surgeon can handle minor bleeding at the same time. Rapid liver parenchymal transection can be performed by thoroughly sharing the roles (Figure 1).

Video 1 provides a narrated summary of the modified two-surgeon technique during laparoscopic liver resection.

**Video 1:** The outline of the modified “two-surgeon technique” in laparoscopic liver resection (with narration).

Mode switching in the two-surgeon technique 

Minimal bleeding can be handled by the secondary surgeon without switching modes during "transection mode." If bleeding from the deep parenchymal fissure occurs, the mode is switched to "hemostatic mode" immediately. To help the secondary surgeon achieve hemostasis, the primary surgeon stretches the surgical field. The surgical field is stretched sufficiently to prevent the bleeding site from becoming a "valley bottom," and the secondary surgeon uses a hemostatic approach appropriate for the bleeding level. The "ball-rolling" movement of saline-linked electrocautery combined (SLiC) can manage most bleeding locations. SLiC's multifaceted ball-rolling approach also stops bleeding from beneath the vein quickly. SLiC's ball-shaped tip is used to apply pressure to the bleeding point from a variety of angles.

Video 2 provides a narrated summary of the role sharing and mode switching in the modified two-surgeon technique during laparoscopic liver resection.

**Video 2:** Role sharing and mode switching in modified two-surgeon technique during laparoscopic liver resection (with narration).

SLiC combined with the wet oxidized cellulose (SLiC-WOC) method in the case of high-output bleeding 

If intractable bleeding is encountered, such as high-output bleeding from the deep parenchymal fissure or major hepatic veins, the saline-linked electrocautery combined with wet oxidized cellulose (SLiC-WOC) approach under the modified two-surgeon technique may be considered [11]. The SLiC-WOC method combines saline-linked electrocautery with wet oxidized cellulose to treat bleeding from the "valley bottom," where the ball cannot reach. In the case of high-output bleeding from the cirrhotic liver, the mode is switched to the "hemostatic mode." The secondary surgeon searches for a bleeding point by using a suction tube to keep the surgical field dry, while the primary surgeon quickly applies the fibrillar form of oxidized cellulose to the surgical field and stretches the field. The secondary surgeon coordinates the operation of the SLiC and suction tube and packs an adequate amount of oxidized cellulose into the bleeding spot. Compression toward the bleeding point and heat coagulation under moderate moisture swiftly accomplish hemostasis (Video 3).

**Video 3:** Saline-linked cautery combined with the wet oxidized cellulose (SLiC-WOC) method for high-output bleeding during laparoscopic liver resection. This video provides an overview of the SLiC-WOC method in the case of high-output bleeding from the cirrhotic liver or the exposed middle hepatic vein.

SLiC-WOC using a modified two-surgeon approach is also beneficial in the case of high-output bleeding from the exposed middle hepatic vein. In this situation, after switching the mode and locating the bleeding point in the middle hepatic vein, an appropriate amount of oxidized cellulose is packed and compressed toward the bleeding point. Under moderate moisture, heat coagulation is applied to achieve hemostasis (Video 3).

Surgical outcome of LLR utilizing a modified two-surgeon technique 

To assess the efficacy and safety of our modified approach to the two-surgeon technique for LLR, surgical outcomes of LLR with the modified two-surgeon technique were compared to those of hybrid liver resection (HLR) (Table 1). Among 162 patients who underwent anatomical liver resection between 2010 and 2019 at our institution, 39 patients underwent LLR utilizing the modified two-surgeon technique (LLR group), and 26 patients underwent HLR utilizing a conventional two-surgeon technique (HLR group). Fisher’s exact probability test and Mann-Whitney U-test were used to compare categorical and continuous variables, respectively. The type of surgery and duration of operation were comparable between the groups, but the occurrence of intraoperative red blood cell transfusion (1% vs. 10%, p < 0.001) or surgical blood loss (70 mL vs. 190 mL, p = 0.018) in the LLR group was significantly lower compared with the HLR group. Concerning the postoperative complications, one gastrointestinal bleeding and one cerebral infarction were experienced in the LLR group, although no bile leakage occurred in either group.

**Table 1:** The comparison of surgical outcomes between patients receiving laparoscopic liver resection utilizing the modified two-surgeon technique and those undergoing hybrid liver resection. HLR: hybrid liver resection; LLR: laparoscopic liver resection; RBC: red blood cell; CD: Clavien-Dindo.

	HLR (n=26)	LLR (n=39)	p value
Type of operation, n (%)			
Sub-segmentectomy	5 (19.2)	12 (30.8)	0.392
Mono-sectionectomy	11 (42.3)	15 (38.5)	0.478
Bi-/Tri-sectionectomy	13 (50.0)	12 (30.8)	0.097
Duration of operation, min, median (range)	304 (254-358)	352 (264-402)	0.071
Intraoperative RBC transfusion, n (%)	10 (38.5)	1 (2.6)	<0.001
Surgical blood loss, mL, median (range)	190 (100-464)	70 (43-160)	0.018
Postoperative morbidity (CD class 3 or higher)	0 (0.0)	2 (5.1)	0.356
Bleeding complication, n (%)	0 (0.0)	1 (2.6)	0.600
Thrombotic complication, n (%)	0 (0.0)	1 (2.6)	0.600
Bile leakage, n (%)	0 (0.0)	0 (0.0)	1.000
Operative mortality, n (%)	0 (0.0)	0 (0.0)	1.000
Length of postoperative stay, d, median (range)	12 (10-14)	8 (7-12)	0.512

## Discussion

This article describes the modified approach of the two-surgeon technique during laparoscopic liver resection. By recognizing and sharing the concepts of "role sharing" and "mode switching," this technique is also feasible and beneficial even when performing liver parenchymal transection laparoscopically.

Yamamoto et al. initially proposed the concept of the two-surgeon technique for open liver resection, known as "Kyoto University-style liver parenchymal resection" [6]. The original procedure included liver parenchymal transection using CUSA by the primary surgeon and meticulous hemostasis using saline-linked bipolar electrocautery by the secondary surgeon. Thereafter, Aloia et al. refined the two-surgeon technique, demonstrating that by allowing two surgeons to participate concurrently in liver parenchymal transection, surgical blood loss, operative time, and the incidence of postoperative bile leakage were significantly reduced [7]. It is, however, difficult to execute these procedures simultaneously when performing liver parenchymal transection laparoscopically. As a result, we modified the approach to make it more appropriate for laparoscopic surgery.

Liver parenchymal transection can be accomplished laparoscopically more quickly and safely with our modified two-surgeon technique. To achieve proper "role sharing," the "transection mode" and the "hemostatic mode" are independent sets in place in this procedure, and these modes are switched rigidly according to the surgical field condition. By thoroughly sharing the roles, satisfactory liver parenchymal transection can be accomplished. The current modified approach provides for rapid liver parenchymal transection and hemostasis during LLR, which is comparable to open liver resection.

The capacity to concurrently handle two tasks, parenchymal transection, and hemostasis, is required for efficient and safe liver resection. Most hepatic parenchymal transections are accomplished using a mix of devices and procedures since no single device has been created that is appropriate for both of these tasks. When conducting the modified two-surgeon technique, we presently employ a ball-tipped SLiC device in soft-coagulation mode, which distributes energy in the radiofrequency range via a constant stream of saline dripping from the device tip [11]. Small arteries and liver parenchyma are coagulated without char production. Because the "ball-rolling" movement of ball-tipped SLiC may be used to provide heat coagulation to the bleeding point from a variety of angles, most bleeding sites can be managed, and the bleeding can be stopped quickly.

In addition, the modified two-surgeon technique during LLR is both safe and advantageous to the training of both young and experienced surgeons as well as the entire surgical team. Surgical team members' LLR abilities may be strengthened early in their careers by teaching them to recognize and discuss the concepts of "mode switching" and "role sharing" during LLR using this method. It is feasible to give enough on-the-job training that is comparable to open surgery by completely sharing the tasks. The plateau phase of the learning curve can be reduced, and the abilities of each surgeon can be enhanced early if new surgeons are regularly exposed to the primary surgeon's functions under the supervision of an attending secondary surgeon. As a result, the surgical team as a whole can increase their abilities. Even young surgeons with little experience learn the method early and contribute to the surgical team's overall success at our facility. Although that LLR has a considerably steeper learning curve than other laparoscopic procedures, the present approach can enable individual surgeons and the entire surgical team to learn more about how to do their jobs better.

## Conclusions

Laparoscopic liver resection using a modified two-surgeon technique is safe and efficient. The concepts of "role sharing" and "mode switching" can be used to accomplish satisfactory liver parenchymal transection and hemostasis. This approach is also beneficial for the training of young surgeons and the surgical team as a whole.
